# Reduced annexin A6 expression promotes the degradation of activated epidermal growth factor receptor and sensitizes invasive breast cancer cells to EGFR-targeted tyrosine kinase inhibitors

**DOI:** 10.1186/1476-4598-12-167

**Published:** 2013-12-19

**Authors:** Rainelli B Koumangoye, Gladys N Nangami, Pamela D Thompson, Vincent K Agboto, Josiah Ochieng, Amos M Sakwe

**Affiliations:** 1 Department of Biochemistry and Cancer Biology, Meharry Medical College, 1005 Dr. DB Todd Jr. Blvd, Nashville, TN 37208, USA; 2 Division of Surgical Oncology & Endocrine Surgery, Vanderbilt University Medical Center, Nashville, TN 37232, USA; 3 Department of Family and Community Medicine, Meharry Medical College, Nashville, TN 37208, USA

**Keywords:** Annexin A6, EGFR, Tyrosine kinase inhibitors, Basal-like breast cancer, Metastasis

## Abstract

**Background:**

The expression of annexin A6 (AnxA6) in AnxA6-deficient non-invasive tumor cells has been shown to terminate epidermal growth factor receptor (EGFR) activation and downstream signaling. However, as a scaffolding protein, AnxA6 may stabilize activated cell-surface receptors to promote cellular processes such as tumor cell motility and invasiveness. In this study, we investigated the contribution of AnxA6 in the activity of EGFR in invasive breast cancer cells and examined whether the expression status of AnxA6 influences the response of these cells to EGFR-targeted tyrosine kinase inhibitors (TKIs) and/or patient survival.

**Results:**

We demonstrate that in invasive BT-549 breast cancer cells AnxA6 expression is required for sustained membrane localization of activated (phosho-Y1068) EGFR and consequently, persistent activation of MAP kinase ERK1/2 and phosphoinositide 3**-**kinase/Akt pathways. Depletion of AnxA6 in these cells was accompanied by rapid degradation of activated EGFR, attenuated downstream signaling and as expected enhanced anchorage-independent growth. Besides inhibition of cell motility and invasiveness, AnxA6-depleted cells were also more sensitive to the EGFR-targeted TKIs lapatinib and PD153035. We also provide evidence suggesting that reduced AnxA6 expression is associated with a better relapse-free survival but poorer distant metastasis-free and overall survival of basal-like breast cancer patients.

**Conclusions:**

Together this demonstrates that the rapid degradation of activated EGFR in AnxA6-depleted invasive tumor cells underlies their sensitivity to EGFR-targeted TKIs and reduced motility. These data also suggest that AnxA6 expression status may be useful for the prediction of the survival and likelihood of basal-like breast cancer patients to respond to EGFR-targeted therapies.

## Background

Annexin A6 (AnxA6), a structurally unusual member of the annexin family of calcium-dependent phospholipid binding proteins, interacts with cellular membranes in a manner that is distinct from other annexins [1]. AnxA6 has also been shown to be down regulated in end-stage heart failure [2], during chronic atrial fibrillation [3] and in malignant forms of melanomas [4]. We recently also showed that AnxA6 is down regulated in breast invasive ductal carcinomas and even more so in breast adenocarcinomas [5].The unifying characteristic of these conditions is that the highly regulated Ca^2+^ entry into cells is uncoupled in cells that either lack, or express low levels of AnxA6. The resulting increase in cytosolic Ca^2+^ in these cells underlies at least in part, the increased contractility of cardiomyocytes [6] and enhanced proliferation of tumor cells [5,7] as well as AnxA6-modulation of tumor cell proliferation, differentiation and motility. While reduced expression of AnxA6 enhances cell proliferation [5,7] lack of or reduced expression of the protein has been shown to be associated with a decrease in the migration of invasive breast cancer cells (BCCs) [5] and chick cranial crest cells [8]. Meanwhile, loss of AnxA6 was associated with a delay in terminal differentiation of murine growth plate chondrocytes due to decreased expression of terminal differentiation markers [9]. This suggests that AnxA6 is a tumor suppressor and a metastasis promoting factor. However, available evidence does not suggests a direct involvement of AnxA6 in these cellular functions. AnxA6 presumably modulates these cellular functions as a scaffolding protein by influencing the localization, expression levels and/or activity of other cellular factors.

The expression of epidermal growth factor receptor (EGFR) in basal-like breast cancer is associated with poor prognosis [10-12] but more importantly, it provides the possibility to therapeutically target the receptor using either tyrosine kinase inhibitors (TKIs) or therapeutic monoclonal antibodies [13,14]. Although EGFR levels are elevated in several cancers, its prognostic and therapeutic significance in various cancers are quite variable. This is presumably due to the association of patient survival with the total receptor rather than the activated receptor levels [15]. It is also possible that the relatively modest EGFR prognostic value in some cancers including breast cancer, may be due to the modulation of its cellular levels and activity by amongst other cellular factors scaffolding proteins such as MUC4 [16] and AnxA6 [17-20].

AnxA6A is largely considered to be a tumor suppressor. This is based on a number of reports that have amply demonstrated that over expression of the protein in the non-invasive A431 epidermoid carcinoma cells as well as BT20 and MDA-MB-468 breast cancer cells that either lack, or express low levels of AnxA6 inhibited their growth [20]. On the other hand, down regulation of AnxA6 in MDA-MB-436 [20] and BT-549 [5] both of which express high levels of AnxA6, led to increased anchorage-independent growth. The inhibition of tumor cell proliferation following the expression of AnxA6 in AnxA6-low cells has been shown to be partly due to the inactivation of activated EGFR and the termination of EGFR-mediated activation of the Ras pathway. These studies revealed that the AnxA6-mediated inactivation of activated EGFR and inhibition of the Ras signaling pathway were respectively mediated via the interaction of AnxA6 with activated protein kinase C (PKC)-α [21] and p120GAP, the Ras-specific guanine nucleotide activating protein [19,22]. The enhanced growth of AnxA6-deficient tumor cells on the other hand is currently believed to be driven by the high cytosolic Ca^2+^-induced activation of PKC isoforms that in turn activate the Ras pathway independently of EGFR activity [20,23]. Besides these findings, other reports have suggested that by acting as a link between the actin cytoskeleton and the plasma and endosomal membranes [7,24,25], its involvement in vesicular transport [26,27] and localization in cholesterol-rich lipid rafts [28,29], AnxA6 on the contrary, contributes to the stabilization of activated receptors on the cell surface [18].

A number of studies have clearly demonstrated that although ligand-activated EGFR is rapidly internalized and degraded in lysosomes [30-32] it can also be recycled back to the plasma membrane [32]. Contrary to its inhibitory effect on EGFR activation and activity in non-invasive tumor cells that either lack, or express low levels of AnxA6 [19,22], we hypothesized that in AnxA6-expressing invasive tumor cells AnxA6 may promote a sustained cell-surface expression of activated EGFR and therefore, persistent receptor activity that drives cell migration. We therefore, investigated the contribution of AnxA6 in the activity of EGFR in invasive breast cancer cells and examined whether the expression status of AnxA6 influences the response of these cells to EGFR-targeted TKIs and/or patient survival. We demonstrate that reduced AnxA6 expression not only promoted rapid degradation of activated EGFR and reduced motility but also sensitized the cells to EGFR-targeted TKIs. We also show that low AnxA6 expression is associated with a better relapse-free survival but poorer overall and distant metastasis-free survival of basal-like breast cancer patients. Together, this demonstrates that the rapid degradation of activated EGFR in AnxA6-depleted invasive tumor cells underlies their sensitivity to EGFR-targeted TKIs and attenuated motility. These data also suggest that AnxA6 expression status may be useful for the prediction of the survival and likelihood of basal-like breast cancer patients to respond to EGFR-targeted therapies.

## Results

### AnxA6 is required for the localization of activated EGFR on the surface of breast cancer cells

**Figure 1:**
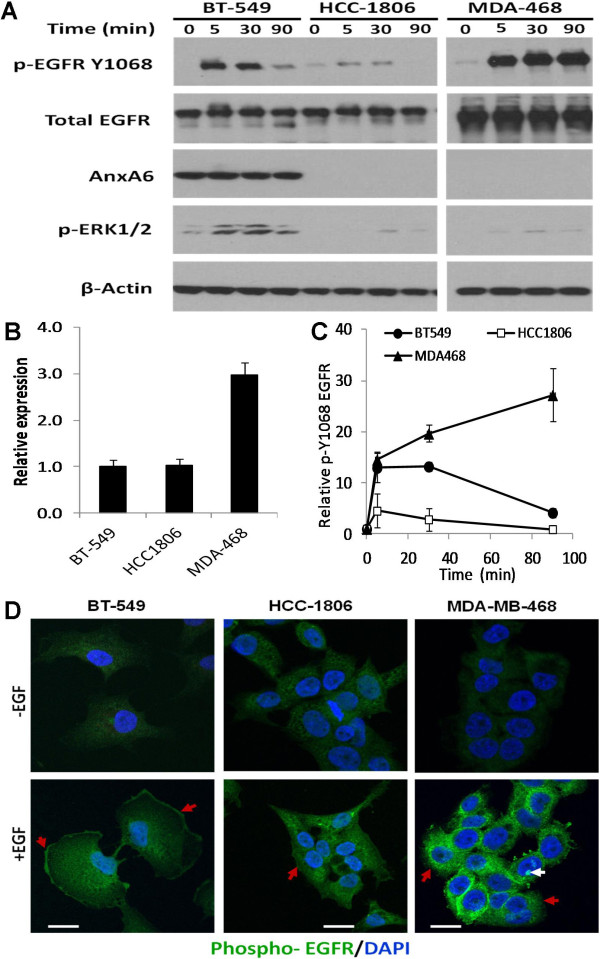
**AnxA6 is required for the localization of activated EGFR on the surface of breast cancer cells. A)** BT-549, HCC1806 and MDA-MB-468 cells were grown to 70% confluency, followed by serum starvation for 24 h. Cells were then treated with EGF for 0–90 min and harvested by scrapping in ice-cold PBS. Equal amounts of whole cell lysates were separated in 4-12% polyacrylamide gels under reducing conditions and analyzed by Western blotting with the indicated antibodies. **B)** Densitometric analysis of EGFR expression. Bars represent EGFR expression relative to BT-549 cells from at least three independent experiments. **C)** Densitometric analysis of EGF-induced EGFR activation. Points represent activated EGFR (p-Y1068) relative to untreated control cells from at least three independent experiments. **D)** AnxA6 expression promotes a sustained cell surface localization of activated EGFR in breast cancer cells. BT-549, HCC1806 and MDA-MB-468 cells were grown on glass cover slips in complete DMEM/F12 medium, then serum-starved overnight. The cells were subsequently washed twice in Hanks’ balanced salt solution (HBSS) and then treated with or without EGF for 5 min. The cells were then fixed for 20 min in 3% paraformaldehyde in PBS. Activated EGFR was detected by immunofluorescence staining with antibodies to phospho-EGFR (Y1068) and counterstained with DAPI. Bars = 10 μm.

It has been amply demonstrated that AnxA6 [28,29] and EGFR [33-35] are components of lipid raft containing membrane microdomains. It has also been shown that activation of EGFR is independent of AnxA6 expression [23], and that intact lipid rafts were required for the activation of the receptor [35]. Together, this led us to speculate that AnxA6 expression is required for sustained cell surface localization of activated EGFR in BCCs. To test this we first sought to compare the activation and activity of EGFR in the invasive AnxA6 high BT-549 cells with that of the non-invasive AnxA6-low HCC1806 as well as MDA-MB-468 cells (with amplified EGFR expression). We show that the expression of AnxA6 is barely detectable in HCC1806 and MDA-MB-468 cells compared to BT-549 cells (Figure 1A). Meanwhile, BT-549 and HCC1806 expressed relatively similar levels of total EGFR while the expression of EGFR was at least 3-fold higher in MDA-MB-468 (Figure 1A and B). Interestingly, treatment of these cells with EGF stimulated to varying extents, the autophosphorylation of the receptor on Y1068 (p-EGFR Y1068). Analysis of the time course for the activation of EGFR revealed that the receptor remained strongly activated even after 90 min in MDA-MB-468 cells. The activated receptor levels however, decreased with time in both HCC1806 and BT-549 cells (Figure 1C). Figure 1A also shows that in the AnxA6-high BT-549 cells, the activation of EGFR led to a sustained activation of MAP kinase ERK1/2 (p-ERK1/2). Paradoxically, in the AnxA6-low HCC1806 and MDA-MB-468 cells and compared to BT-549 cells, EGFR activation led to relatively reduced activation of ERK1/2 (Figure 1A).

To explain this paradox, we examined the localization of the activated receptor in the three cell lines by immunofluorescence. As shown in Figure 1D, there was a robust EGF-stimulated activation of EGFR in the AnxA6-low MDA-MB-468 cells. Interestingly, the activated EGFR in these cells was essentially localized to the perinuclear/cytoplasmic regions (red arrow heads) and in some cells, sequestered into the nucleus (white arrow head). Similarly, and consistent with Figure 1A, in the AnxA6-low HCC1806 cells, plasma membrane-localized activated EGFR (red arrow head) was also barely detectable (Figure 1D). On the contrary, in the AnxA6-high BT-549 cells, the activated EGFR was predominantly localized to the plasma membrane (red arrow heads). These data suggest that AnxA6 enhances the localization of activated EGFR on the cell surface and that this is accompanied by sustained activation of down-stream effectors such as ERK1/2.

**Figure 2:**
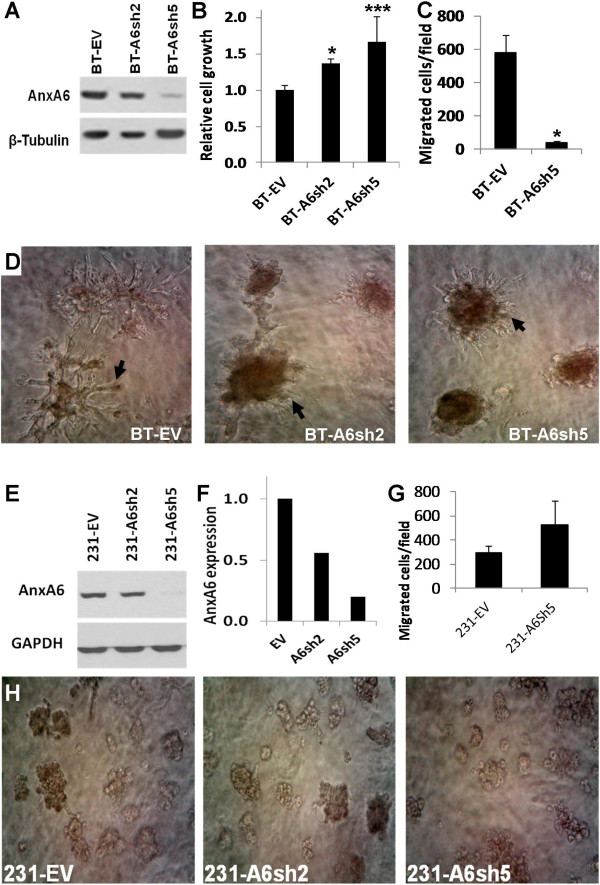
**Depletion of AnxA6 in invasive breast cancer cells promotes cell proliferation but inhibits cell motility. (A)** AnxA6-depleted BT-549 cell lines were generated as described in “Materials and methods”. Whole cell lysates from the selected clones designated A6sh2 and A6sh5, were analyzed by Western blotting. **(B)** Cell proliferation assays. Cells were plated in quadruplicate in 24 well plates and cultured for 72 h in complete DMEM/F12 medium. Cell growth and viability was assayed using PrestoBlue reagent. Bars represent cell growth ± S.E.M from three independent determinations. * p < 0.05; *** p < 0.001. **(C)** Migration assays. Serum-starved AnxA6-depleted BT-549 and control cells were plated in duplicate in the upper chambers of 8 μm culture inserts. Complete DMEM/F12 was used as the chemoattractant. Bars represent the number of migrated cells/field ± s.d from three independent determinations. * p < 0.001. **(D)** Anchorage-independent growth of AnxA6-depleted BT-549 cells. Control (BT-EV) and AnxA6-depleted (BT-A6sh5 and BT-A6sh2) cells were maintained in matrigel 3D cultures for up to 10 days. Digital images of the colonies were captured with a digital camera (x10 magnification). **(E-H)** Analyses of AnxA6-depleted MDA-MB-231 cells. The expression levels of AnxA6 **(E)**, Densitometric analysis of AnxA6 expression **(F)**, cell migration **(G)** and growth in 3D cultures **(H)** were examined as described in **A-D** above.

To further ascertain the requirement of AnxA6 in the sustained localization of activated EGFR at the cell surface, and whether this is required for the invasiveness of these cells, we used RNA interference to down-regulate AnxA6 in the AnxA6-high BT-549 cells. Two AnxA6-depleted cell lines designated BT-A6sh2 (clone A1) and BT-A6sh5 (clone A3) were selected from 10 different clones (Additional file 1: Figure S1) and respectively showed ~30% and >80% AnxA6 depletion by Western blotting (Figure 2A). The AnxA6-depleted cells grew more efficiently than the control cells (Figure 2B) and as previously shown [5], their motility was significantly inhibited (Figure 2C). As shown in Figure 2D AnxA6 depletion also induced a transformation from invasive stellate colony morphology with long invasive projections in control BT-549 cells to the non-invasive acinar-like colony morphology in BT-A6sh5 cells. Invasive projections in BT-A6sh5 cells if discernible were much shorter than those in control cells, suggesting loss of invasiveness. This change in colony morphology is dependent on AnxA6 expression level because BT-A6sh2 cells showed intermediate colony morphologies. Based on these data, the BT-A6sh5 cell line with the most AnxA6 depletion was used as the AnxA6-depleted cell line in most of the following experiments. Interestingly, a similar extent of AnxA6 down regulation in MDA-MB-231 cells did not substantially lead to altered colony morphology and if anything, the cells tended to be more motile than the control cells (Figure 2E-H).

To substantiate the different outcomes of AnxA6 depletion in BT-549 and MDA-MB-231 TNBC cell lines on cell growth and motility, we examined the expression of AnxA6 and EGFR genes in these and other breast cell lines by qRT-PCR. This analysis revealed that MDA-MB-231 cells express relatively reduced levels of both AnxA6 and EGFR compared to BT-549 cells (Additional file 2: Figure S2A and B). We also demonstrate that the response of MDA-MB-231 to EGF treatment is also different from that in BT-549 cells in that while PI3 kinase/Akt and MAP kinase ERK1/2 are strongly activated in BT-549 cells, Akt and to a lesser extent ERK1/2 activation in MDA-MB-231 cells are relatively attenuated (Additional file 2: Figure S2B). As previously reported in other AnxA6-deficient tumor cells [20], over expression of AnxA6 in HCC1806 cells on the other hand was associated with reduced activation of the receptor and ERK1/2 (Additional file 3: Figure S3A-D). AnxA6 expression in HCC1806 cells also inhibited their growth in 3D cultures (Additional file 3: Figure S3E). These data suggest that in triple negative breast cancer cells, the modulation of EGFR activation and/or activity by AnxA6 is not only dependent on the AnxA6 expression levels but is also cell type specific.

### Reduced AnxA6 expression promotes the degradation of Activated EGFR

**Figure 3:**
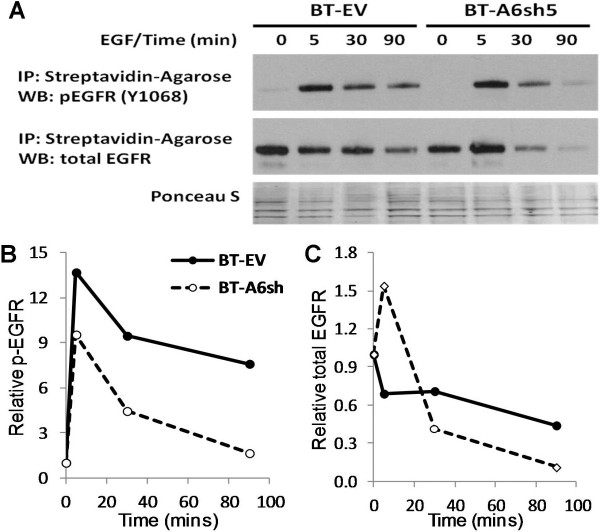
**Down regulation of AnxA6 in invasive breast cancer cells enhances the degradation of cell surface-associated EGFR. (A)** AnxA6 depleted (BT-A6sh5) and control (BT-EV) cells were treated with EGF for 0–90 min and the cell surface-associated proteins were biotinylated as described in “materials and methods”. The biotinylated proteins were isolated from whole cell lysates using Streptavidin-agarose beads and analyzed by Western blotting using antibodies to activated and total EGFR. Ponceau S staining of the blots was used as loading control. **(B and C)** Densitometric analysis of residual cell surface-associated activated EGFR **(B)** and cell surface-associated total EGFR **(C)**. Points represent residual biotin-labeled cell surface-associated EGFR in the control (solid lines with closed circles) and AnxA6-depleted cells (broken lines with open circles) at the indicated times from a representative experiment. IP = immunoprecipitation; WB = western blot.

The desensitization of ligand activated EGFR like most cell surface receptors predominantly occur by rapid internalization of receptor-ligand complexes and degradation in lysosomes. Given the strong cell surface expression of activated EGFR in AnxA6 expressing BT-549 cells, we speculated that the virtually absent expression and attenuated activity of the receptor in the AnxA6-low HCC1806 and MDA-MB-468 cells could be attributed to the fate of the activated receptor. To verify this, BT-549 control or AnxA6-depleted cells were treated with or without EGF for 0–90 min, surface biotinylated and the fate of EGFR examined by western blotting. Assessment of the residual levels of biotinylated surface-associated total and pY1068-EGFR in control (AnxA6-high) and AnxA6-depleted (AnxA6-low) BT-549 cells revealed that EGFR activation per se was indeed unaffected by AnxA6 depletion (Figure 3A). As expected, the levels of remaining ligand-activated EGFR decreased with time in both cell lines. However, the residual cell surface-associated activated EGFR decreased more rapidly in AnxA6-depleted cells (BT-A6sh5) compared to that in control cells (Figure 3A and B). By 90 min 60% of the activated EGFR in control cells was still cell surface-associated compared to only 20% in AnxA6-depleted cells. Similarly, the decrease in total cell surface (biotinylated) EGFR in the control cells was initially more rapid but this continued more slowly thereafter. On the contrary, there was a transient delay in the down-regulation of biotin-labeled EGFR that was followed by a more rapid decrease in the cell surface EGFR levels (Figure 3A and C). Within 90 min of EGF treatment, the receptor in AnxA6-depleted cells decreased to about 10% compared to about 40% in control cells. Taken together and consistent with data in Figure 1D, these data reveal that AnxA6 depletion in invasive breast cancer cells was accompanied by a rapid decrease in the total and activated cell surface EGFR levels. Furthermore, the transient delay in AnxA6-depleted cells versus a rapid initial decrease in the levels in control cells suggests a role of AnxA6 in the internalization and/or trafficking of the activated receptor.

**Figure 4:**
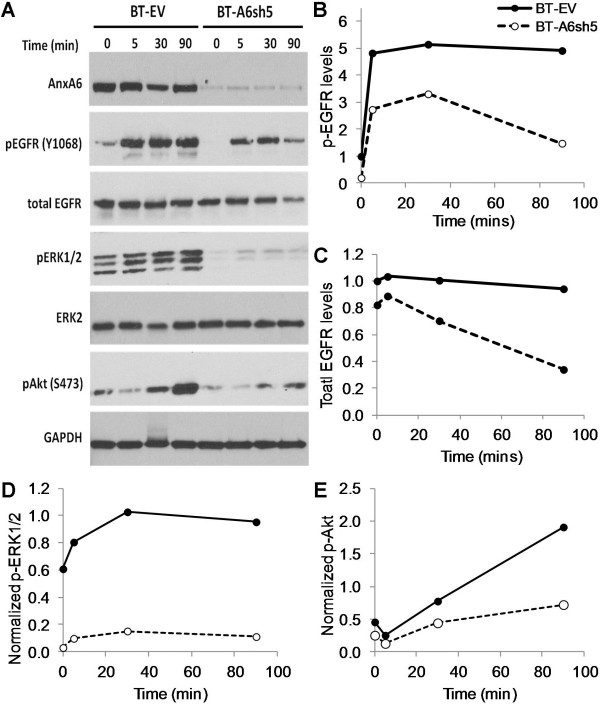
**Effects of AnxA6-depletion on the dynamics of total cellular EGFR. A)** AnxA6-depleted (BT-A6sh5) and control (BT-EV) BT-549 cells were treated with EGF for 0–90 min and whole cell lysates were analyzed by Western blotting to determine the residual activated and total EGFR. **(B-E)** Densitometric analysis of residual activated EGFR **(B)**, total EGFR **(C)** phospho-ERK1/2 **(D)** and phospho-Akt **(E)** in BT-EV (solid lines with dark circles) and BT-A6sh5 (broken lines with open circles). Points represent whole cellular EGFR/activated EGFR remaining at the indicated times or activated ERk1/2 and Akt in the control cells (closed circles) and AnxA6-depleted cells (open circles) from a representative experiment.

We next sought to determine whether the rapid decrease in the activated cell surface EGFR in AnxA6-depleted cells and/or the relatively minimal activation of ERK1/2 in either HCC1806 or MDA-MB-468 cells could also be attributed to the lack of or relatively low levels of AnxA6. To do this we examined the residual levels of total EGFR in the AnxA6-depleted and control BT-549 cells. This analysis revealed that the EGF-activated (Figure 4A and B) and the total cellular receptor levels (Figure 4A and C) in control cells remained relatively constant while the receptor levels in AnxA6-depleted cells were not only lower (by at least 3-fold after 90 min), but also decreased more rapidly with time. Densitometric analysis of EGF-stimulated activation of ERK1/2 (Figure 4D) and Akt (Figure 4E) also reveal that these downstream targets were strongly inhibited in the AnxA6-depleted BT-549 cells compared to control cells. Together with data in Figure 3, this suggests that AnxA6 is necessary for the stabilization of the receptor on the cell surface and correspondingly, sustained signaling to downstream effectors (ERK1/2 and Akt).

**Figure 5:**
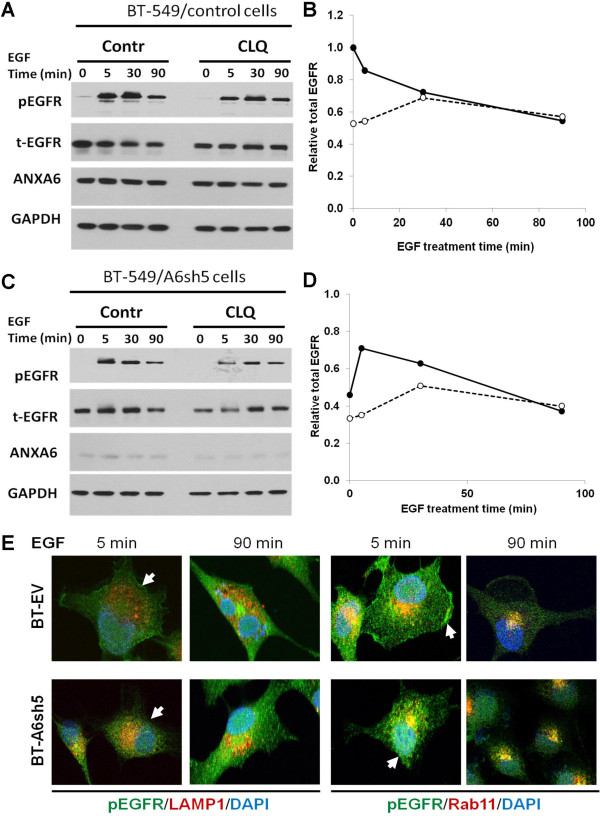
**Effects of AnxA6-depletion on lysosomal degradation and recycling of EGFR. A-D)** Control BT-EV **(A and B)** or AnxA6-depleted BT-A6sh5 **(C and D)** cells were pretreated overnight with or without CLQ (50 μg/ml) during serum-starvation. Cells were briefly rinsed in HBSS, treated with EGF for up to 90 min in the presence or absence of CLQ respectively. Total and Y1068-EGFR were examined by western blotting. **E)** Serum-starved AnxA6-depleted (BT-A6sh5) and control (BT-EV) cells grown on glass cover slips were treated with EGF for 5 or 90 min. The cells were fixed in 3.7% PBS-buffered formaldehyde and processed for indirect immunofluorescence as described in Figure 1D using anti-pY1068 EGFR (green) and either LAMP1 or Rab11 (red). Nuclei were stained with DAPI.

To demonstrate that reduced AnxA6 expression enhanced EGFR degradation, control and AnxA6-depleted BT-549 cells were serum-starved overnight in the presence or absence of chloroquine (CLQ). The cells were then treated with or without EGF and the residual total and activated EGFR were examined by western blotting. As shown in Figure 5A-D, although CLQ treatment did not abolish the degradation of the activated receptor, the total cellular levels of receptor in the control and AnxA6-depleted BT-549 cells were stabilized by CLQ treatment. Interestingly, the levels of the receptor (t-EGFR) in AnxA6-depleted cells were restored to those in the control cell by 90 min (Figure 5A and C). To verify whether there were discernible differences in the degradation and recycling of the activated receptor in the control and AnxA6-depleted BT-549 cells, we examined the co-localization of EGF-activated EGFR with either LAMP1 (a late endosomal marker) or Rab11 (a recycling endosomal marker). As depicted in Figure 5E, within 5 min of EGF treatment, cell surface activated EGFR was clearly discernible in the control cells but the cell surface expression was lost by 90 min (arrows). On the contrary, even within 5 min of EGF treatment, most of the activated EGFR was intracellular in AnxA6-depleted cells (Figure 5E, arrows). We also observed a greater extent of co-localization of activated EGFR with LAMP1 in the AnxA6-depleted BT-549 cells compared to the control cells. Meanwhile, the activated receptor co-localized with Rab11 in both the control and AnxA6-depeleted cells. Together these data suggest that activated EGFR is actively recycled in these cells and that EGFR degradation is enhanced in the AnxA6-depeleted cells.

### AnxA6-depleted invasive breast cancer cells are sensitive to EGFR tyrosine kinase inhibitors

**Figure 6:**
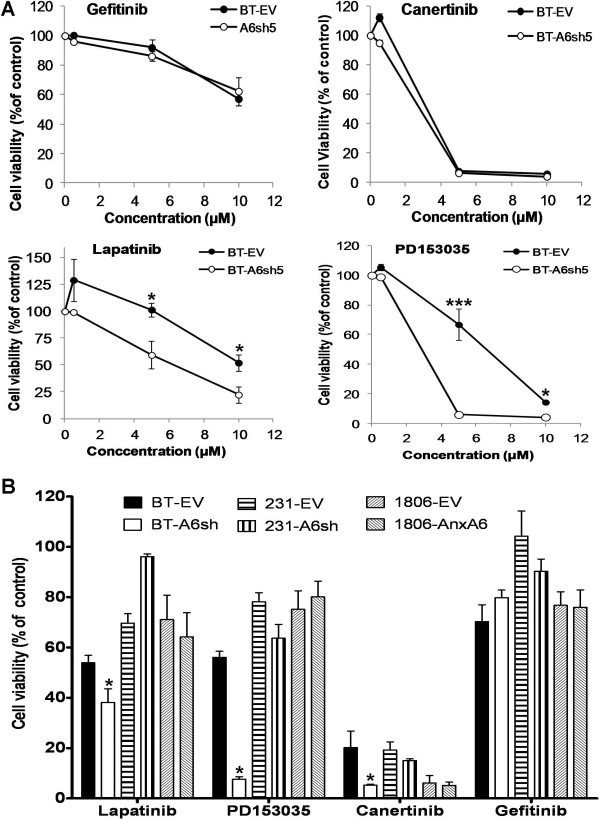
**Down regulation of AnxA6 sensitizes invasive breast cancer cells to EGFR-targeted TKIs. A)** Control (BT-EV) and AnxA6-depleted (A6sh5) BT-549 cells were cultured as above and treated with the indicated concentrations of the indicated EGFR TKIs for 72 h. Cell viability/proliferation was determined using PrestoBlue cell viability assay. Experiments were performed in triplicate and repeated at least three times. Points represent mean viability ± S.E.M relative to DMSO control treated cells. * p < 0.05, *** p < 0.0001 versus respective controls. **B)** AnxA6-depleted MDA-MB-231 and BT-549 as well as AnxA6-over expressing HCC1806 cells with their respective controls were treated with 5 μM of the indicated EGFR-TKI or DMSO control. Cell viability was determined as above. Bars represent mean viability ± S.E.M relative to DMSO control treated cells. * p < 0.05 versus respective controls.

Given that down regulation of AnxA6 in invasive breast cancer cells was accompanied by a decrease in the total and activated EGFR in invasive breast cancer cells, we speculated that AnxA6-depletion in these cells might affect their response to EGFR-targeted TKIs. To explore this further, AnxA6-depleted and control BT-549 cells were treated with various concentrations (0–10 μM) of EGFR-targeted TKIs for 72 h. Figure 6A shows that gefitinib (Iressa) was the least potent of the four compounds while canertinib (CI-1033) was the most potent. In a concentration-dependent manner, AnxA6-depleted BT-549 cells were more sensitive to lapatinib (Tykerb) and PD153035 (Tyrphostin, AG 1517) compared to control cells. As shown in Table 1, the IC50s for inhibition of cell growth for lapatinib and PD153035 were significantly lower in AnxA6-depleted cells compared to control cells. This suggests that reduced AnxA6 sensitizes invasive BCCs to some EGFR-targeted TKIs.

**Table 1:** IC50 values for EGFR-targeted TKIs in control and AnxA6-depleted invasive breast cancer cells

***EGFR-targeted TKI***	***BT-549-EV***	***BT-549-A6sh***	***Fold change EV:A6sh***	***P-value EV:A6sh***
**PD153035**	6.46 ± 0.62	4.16 ± 0.12	1.55	0.02*
**Lapatinib**	14.09 ± 1.75	5.99 ± 1.77	2.35	0.03*
**Canertinib**	4.30 ± 0.24	3.96 ± 0.11	1.09	0.10
**Gefitinib**	14.43 ± 0.71	13.51 ± 2.83	1.07	0.80

IC50 - half maximal inhibitory concentration (μM) estimated from the regression plots for the indicated tyrosine kinase inhibitors (TKI). BT-549-EV, control BT-549 cells; BT-549-A6sh, AnxA6-depleted BT-549 cells. For fold change and p-values, EV: A6sh denotes, BT-549 control cells versus AnxA6-depleted cells. *Statistically significant at p < 0.05.

To validate the above data, we compared the response to these compounds of AnxA6-depleted MDA-MB-231 and BT-549 cells as well as AnxA6-over expressing HCC1806 cells with their respective controls. As depicted in Figure 6B, treatment of these cells with these compounds (5 μM) or DMSO control confirmed that while AnxA6 depletion in the invasive BT-549 cells significantly sensitized the cells to lapatinib, PD153035 and canertinib, AnxA6-depletion in MDA-MB-231 cells did not significantly alter their sensitivity to these compounds. Meanwhile, over expression of AnxA6 in HCC1806 did not alter their response to these EGFR-targeted TKIs. Together these data confirm the variable response of breast cancer cells to EGFR targeted therapies suggest that reduced AnxA6 expression may be more relevant in breast cancer subtypes such as EGFR expressing invasive basal-like breast cancer.

### AnxA6 expression status is associated with the survival of patients with basal-like breast cancer

**Figure 7:**
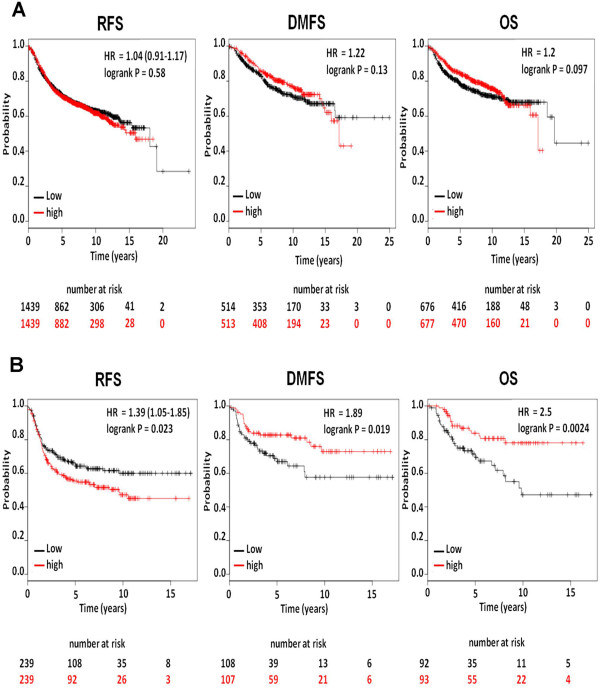
**Survival analyses of patients with high and low AnxA6-expressing breast cancers.** Kaplan Meier plots showing the relationship between high or low AnxA6 expression and recurrence-free (RFS), distant metastasis-free (DMFS) and overall survival (OS) of **(A)** all breast cancer patients combined and **(B)** basal-like breast cancer patients. The Kaplan-Meier plots and the number of patients at risk at given time points are indicated for low AnxA6 expression status (black) and for high AnxA6 expression status (red).

To support the above conclusion, we examined whether AnxA6 expression status also influences the survival of breast cancer patients with varied clinical disease. To do this we used the KM plotter, a recently reported publicly available online survival analysis tool [36] that has been used extensively to analyze the expression of 22,277 genes on the survival of 2,977 breast cancer patients [37-39]. This analysis revealed that, AnxA6 expression status is not associated with the overall (OS), relapse-free (RFS) or distant metastasis-free (DMFS) survival of all breast cancer patients combined (Figure 7A). AnxA6 expression status also is not associated with the survival of patients with luminal breast cancer or those with different HER2, estrogen or progesterone receptor status. However, AnxA6 expression status is significantly associated with the survival of patients with basal-like breast cancer. As shown in Figure 7B, low AnxA6 expression is associated with a significantly higher RFS for patients with basal-like breast cancer (p = 0.023). High AnxA6 levels were on the other hand, are associated with higher OS (p = 0.0024) and DMFS (p = 0.019) for patients with this breast cancer subtype. A previous evaluation of multiple studies with more than 20,000 patients also showed that high EGFR expression is associated with lower RFS for patients with head and neck, ovarian, cervical, bladder and oesophageal cancers [15]. However, the prognostic value of EGFR expression in other cancers including breast cancer was found to be modest [15].

## Discussion

Two opposing notions have thus far emerged from several studies on the potential functions of AnxA6 in cancer progression. On one hand, AnxA6 has been shown to terminate EGFR activity by not only inhibiting the activation of the receptor but also by inhibiting EGFR-induced activation of the Ras pathway [20,23]. This is supported by the inhibition of anchorage-independent growth following over expression of AnxA6 in cells that either lack, or express low levels of the protein [20,23]. On the contrary, there is ample evidence suggesting that AnxA6 contributes to the stabilization of activated receptors on biological membranes that eventually leads to sustained downstream signaling. However, the involvement if any, of AnxA6 in the stabilization of EGFR on the cell surface is as yet unclear. In the present study we showed that AnxA6 is indeed required for the sustained localization of activated EGFR on the surface of invasive tumor cells and that this contributes to persistent activation of downstream effectors that drive motility and invasiveness of the cells. This notion is supported by the rapid degradation of activated EGFR, loss of invasiveness and sensitivity to EGFR-targeted TKIs following down regulation of AnxA6 in invasive breast cancer cells. Meanwhile the enhanced proliferation of cells that lack or express low levels of AnxA6 has been shown to be mediated by PKC activation of the Ras pathway independently of EGFR activity [20,23]. Together, these data suggest that while stabilization of activated EGFR by AnxA6 may be important in the dissemination of invasive tumor cells, EGFR activity is dispensable in the enhanced proliferation of cells that either lack, or express low levels of AnxA6. Conflicting data were however, observed following down regulation of AnxA6 in the AnxA6-high BT-549 cells and in MDA-MB-231 cells that expressed comparatively lower levels of the proteins. Given the heterogeneity of breast cancer cells, it is plausible to suggest that the different outcomes of AnxA6 modulation of EGFR in MDA-MB-231 cells and BT-549 cells are cell type specific and presumably dictated by the level of AnxA6 expression.

It is well established that activated EGFR is endocytosed and either degraded in lysosomes or recycled back to the cell surface [30,32,40]. It has also been shown that receptors localized at the cell surface are more efficient in eliciting downstream signaling than those localized in endocytic compartments [41,42]. The strong activation of EGFR in the AnxA6-low MDA-MB-468 cells with relatively reduced activation of ERK1/2 is consistent with the localization of activated EGFR in the perinuclear/endocytic compartment as opposed to plasma membrane localized activated EGFR. The plasma membrane localization of activated receptor correlates with robust activation of ERK1/2 in the AnxA6-high BT-549 cells. From previous studies that demonstrated that both EGFR [33,35,43] and AnxA6 [17,44,45] are components of lipid rafts and that EGFR activation occurs in lipid rafts [35], it appears that the AnxA6-dependent membrane stabilization of activated EGFR occurs mainly in lipid rafts. Therefore, the low levels of activated EGFR on the surface of MDA-MB-468 or HCC1806 cells and the reduced levels of activated receptor in AnxA6-depleted BT-549 cells may be attributed to the absence or disruption of AnxA6-stabilized lipid rafts in these cells.

The development of resistance to TKIs is common and represents a major impediment to targeted treatments with these compounds. A recent study demonstrated that localization of EGFR in lipid rafts enhanced the resistance of tumor cells to gefitinib [33]. Consistent with this report, we also showed that AnxA6-depleted BT-549 cells were more sensitive to lapatinib and PD153035 EGFR-targeted TKIs. This increase in the response of AnxA6-depleted cells to EGFR-targeted TKIs may be attributed to the disruption of AnxA6-stabilized lipid rafts and the accompanying instability of activated EGFR. Although further studies are warranted, AnxA6 expression status may underlie a novel mechanism for the development of resistance to EGFR-targeted therapies. Pending validation, patients with the more aggressive basal-like breast cancers in which AnxA6 expression is low may be more likely to respond to some EGFR-targeted therapies.

The growing evidence that AnxA6 expression promotes cell migration but attenuates cell proliferation [5,7] implies that this tumor suppressor plays an important role in breast cancer progression and/or patient survival. This also suggests that AnxA6 expression is associated with cell motility while reduced AnxA6 expression is associated with enhanced tumor cell growth. Given that AnxA6 expression is lower in breast cancer, it was necessary to assess whether AnxA6 expression status is associated with patient outcome. We provide evidence suggesting that reduced AnxA6 expression is significantly associated with higher recurrence-free but lower distant metastasis-free (DMFS) and overall survival of patients with basal-like breast cancer. Basal-like breast cancers are highly aggressive cancers with early patterns of relapse and metastasis to visceral organs [46]. The association of AnxA6 expression status with the survival of patients with this breast cancer subtype is consistent with the modulation of the stability of activated EGFR in invasive breast cancer cells by AnxA6.

Analysis of more than 200 studies involving more than 20,000 patients revealed that the expression of EGFR was also associated with reduced recurrence-free survival in patients with head and neck, ovarian, cervical, bladder and oesophageal cancers. However, EGFR expression was found to exert only a modest prognostic value in other cancers including breast cancer [15]. The low prognostic value was suggested to be due to the correlation of patient survival with the total rather than the activated receptor [15]. It is also possible that this is due to the fact that EGFR activation and activity are modulated by other cellular factors including scaffolding proteins such as MUC4 [16] and AnxA6 [17-20]. The poorer distant metastasis-free and overall survival of patients with low AnxA6-expressing basal-like breast cancers could be attributed to the potentially more aggressive growth of the tumors and/or secondary lesions. On the other hand, and as demonstrated in this study, the better recurrence-free survival of low AnxA6-expressing basal-like breast cancer patients may be due to the their greater probability to respond to targeted therapy.

## Conclusions

In summary, our study is the first to describe an association between a member of the calcium-dependent membrane binding annexin family and survival of patients affected by the highly aggressive basal-like breast cancer. This study demonstrates that the enhanced degradation of activated EGFR in AnxA6-depleted invasive breast cancer cells underlies their sensitivity to EGFR-targeted TKIs and attenuated motility. Further studies are warranted to provide a rationale for the use of AnxA6 expression status as a predictive marker for basal-like breast cancer and to identify patients with this breast cancer subtype who are more likely to respond to EGFR-targeted therapies.

## Methods

### Cell lines and cell culture

The following breast cancer cell lines BT-549, MDA-MB-231 (MDA-MB-231), HCC1806 were purchased from American Type Culture Collection (ATCC). MDA-MB-468 (MDA-MB-468) cells were kindly provided by Dr Ann Richmond, Vanderbilt Medical Center (Nashville, TN). These cell lines were cultured in DMEM/F12 containing 10% FBS, penicillin (100 units/ml), and streptomycin (50 units/ml). Cells were maintained at 37°C in a humidified CO_2_ incubator. Where indicated, serum-starvation of cells was achieved by culturing the cells in DMEM/F12 containing 0.5% FBS for 24 to 48 h.

### Antibodies and other reagents

Antibodies against EGFR, phospho-EGFR (pY1068), phospho-extracellular signal-regulated kinase 1/2 (pERK1/2, T202/Y204) and phospho-Akt (pS473) were purchased from Cell Signaling Technology (Beverly, MA, USA). Antibodies against ERK2, Akt1, GAPDH and AnxA6 were purchased from Santa Cruz Biotechnology, Inc. Antibodies specific for the flag-epitope (anti-flag M2), β-tubulin and β-actin were obtained from Sigma. Except otherwise indicated, secondary anti-mouse, anti-goat and anti-rabbit horseradish peroxidase-conjugated antibodies were purchased from Santa Cruz Biotechnology, Inc or Sigma. EGFR Tyrosine Kinase Inhibitor Set including canertinib, erlotinib hydrochloride, gefitinib, lapatinib ditosylate and PD153035 hydrochloride was purchased from BioVision Inc. Sulfo-NHS-biotin and protease inhibitor cocktail were products from Sigma.

### Plasmid constructs and transfections

Small hairpin RNAs (shRNAs) targeting the coding sequence of AnxA6 in pGIPZ lentiviral vector, a non-silencing shRNA control or the empty vector (Open Biosystems Inc.), were used to transfect BT-549 and MDA-MB-231 BCCs using Lipofectamine 2000 (Invitrogen). The cells were selected with puromycin 48 h post-transfection for up to three weeks. Cells expressing high levels of GFP were then sorted by flow cytometry, cloned by limiting dilution, and the isolated clones were expanded in selection medium containing puromycin (2 μg /ml). The following shRNA sequences were used to target AnxA6 in BT-549 and MDA-MB-231 cells: *A6sh2*, 5'- TTCAGCATTGGTCCGAGTG-3'; *A6sh5*, 5'-TGTGTCTTCGTCAGTCCCG-3'. For experiments involving over expression of AnxA6, the coding sequence of AnxA6 variant 1 (accession No. NM_001155.4) was amplified from plasmid pCMV-Sport6-AnxA6 (Open Biosystems Inc.). The fragment was cloned into Hind III and Xho I linearized pCMV-3Tag-8 (Clontech), and the construct used to transfect the AnxA6-low HCC1806 BCCs using Fugene6 transfection reagent (Promega). The transfected cells herein designated 1806-Anx6 and 1806-EV were selected with hygromycin, cloned as above and expanded in continued hygromycin (100 μg/ml) selection.

### Immunofluorescence microscopy

Cells were plated sparsely on glass cover slips and allowed to grow until they formed colonies of a few cells. Cells were then serum-starved overnight and treated with or without EGF for 5 min. Indirect immunofluorescence staining was performed as previously described [5] using pEGFR (Y1068) antibodies and FITC-conjugated secondary antibodies. Cover-slips were mounted with ProLong Gold antifade containing DAPI (Invitrogen). Images were captured using a Nikon A1R confocal microscope with 60× oil-immersion objectives and analyzed using the NIS software.

### Cell-surface biotinylation and Western blotting

Cells were grown in complete medium until they were 70% confluent, then serum-starved for 24 h and treated with or without EGF for the indicated time points. Following treatment, cells were washed twice with ice-cold phosphate-buffered saline (PBS) pH 7.4, with 0.5 mM Ca^2+^ and 1 mM Mg^2+^) and then incubated with Sulfo-NHS-biotin (0.2 mg/ml in cold PBS) for 30 min at 4°C. Unreacted biotin was quenched with ice-cold 100 mM glycine in PBS for 15 min at 4°C. Whole cell extracts were prepared in TNE lysis buffer (150 mm NaCl, 10 mm Tris–HCl pH 8.0, 1 mm EDTA, and 1% Nonidet P-40 with protease and phosphatase inhibitors). Biotinylated (cell surface) proteins isolated using Streptavidin-agarose beads and whole cell extracts were used for the detection of cell surface and total cellular EGFR respectively by Western blotting as previously described [5]. Immuno-reactive bands were visualized by enhanced chemiluminescence (ECL; Pierce) and quantified using NIH Image J software.

### Activation of EGFR and downstream signaling assays

Breast epithelial and BCCs were cultured until they were 70% confluent then serum-starved overnight and treated with 50 ng/ml EGF (EMD Biosciences) in Hank’s balanced salt solution (HBSS) for the indicated time periods. The EGF treated cells were scraped in ice-cold PBS and total cell lysates prepared as described previously [5]. EGFR activation was detected by immunoblotting with anti-EGFR (pY1068) and antibodies to total EGFR. Activation of downstream signaling cascades was determined by Western blotting using ant-pErk1/2 (pT202/pY204) and anti-pAkt (S473). Immunoblotting with antibodies to either anti-Erk2, anti GAPDH or anti-β-tubulin were used as the loading controls. Immuno-reactive bands were revealed by ECL, scanned and quantified using NIH Image J software. Activation levels were determined as the ratios of phosphoprotein to the total protein or loading controls.

### Cell proliferation assays

The effects of AnxA6 depletion and TKIs on cell growth were performed in 24-well plates in triplicates using 1 x 10^4^ cells/well, as previously described [5]. The proliferation and viability of the cells were determined by incubating the cells in 1:10 diluted PrestoBlue reagent (Invitrogen) in HBSS supplemented with 1 mM Ca^2+^ and 0.5 mM Mg^2+^ for 2–4 h. Cell proliferation was determined by fluorescence measurement following excitation at 540 nm and emission at 600 nm.

### Growth in 3D cultures and motility assays

Clonogenic and motility assays were performed in duplicate as previously described [5]. Digital images of the 3D cultures were captured at x10 magnification using DCM200 digital camera and Scopephoto software. For motility assays, cells were counted from at least 5 separate fields per insert.

### *In silico* analyses

The online KM plotter was used to compare the impact of AnxA6 expression on the survival of 2,977 breast cancer patients according to the set parameters [36]. In order to analyze the prognostic value of a particular gene, the cohorts are divided into two groups according to the median (or upper/lower quartile) expression of the gene. A survival curve is displayed, and the hazard ratio with 95% confidence intervals and logrank P value are calculated and displayed [36]. We tested the effect of high or low AnxA6 expression on the overall, distant metastasis-free and recurrence-free survival of either all patients or patients with various breast cancer molecular subtypes.

### Statistical analysis

Data were analyzed using Microsoft Excel 2007. Except otherwise indicated data were presented as mean ± SD. Data were analyzed using Student’s t-test; a p-value < 0.05 was considered statistically significant.

## Abbreviations

AnxA6: Annexin A6; BCCs: breast cancer cells; BLBC: basal-like breast cancer; TNBC: triple negative breast cancer; EGFR: epidermal growth factor receptor; TKI: tyrosine kinase inhibitors; OS: overall survival; RFS: relapse-free survival; DMFS: distant metastasis-free survival.

## Competing interests

The authors declare that they have no competing interests.

## Authors’ contributions

RK, was responsible for the execution, data interpretation and data analyses; GN and PT were responsible for cell line maintenance; VA and AS were responsible for the KM survival analyses; JO contributed to experimental design and editing of the manuscript. AS directed the experimental design and provided insight for experimental execution, and drafting of the manuscript and figures. All authors have read and approved the final manuscript.

## Supplementary Material

Additional file 1: Figure S1 Down regulation of AnxA6 in BT-549 and MDA-MB-231 breast cancer cells. (A) BT-549 and MDA-MB-231 cell lines were transfected with shRNAs in pGIPZ and cloned as described in “Materials and methods”. Whole cell lysates from the isolated clones generated from two distinct shRNAs designated A6sh2 and A6sh5 were analyzed by Western blotting. Densitometric analysis of the expression of AnxA6 expression relative to GAPDH is presented. B) Whole cell lysates from the selected clones were analyzed by western blotting using antibodies against AnxA6, EGFR and GAPDH. Click here for file

Additional file 2: Figure S2 Differential expression of AnxA6 and EGF-induced activation of EGFR in normal breast epithelial and breast cancer cells A) mRNA levels of AnxA6 and EGFR in normal breast epithelial and breast cancer cells. Equal amounts (1 μg) of total RNA extracted from the indicated cell lines were used for the first strand synthesis and quantitative PCR was programmed with 10% of the first strand reactions. Bars represent gene expression levels normalized to GAPDH ± s.d. from three independent determinations. (B) AnxA6 expression and EGF-induced activation of EGFR and downstream signaling in normal and breast carcinoma cell lines. The indicated cell lines were grown to 70% confluency, followed by serum starvation for 24 h. Cells were then treated with EGF for 0–90 min and harvested by scrapping in ice-cold PBS. Equal amounts of whole cell lysates were separated in 4-12% polyacrylamide gels under reducing conditions and analyzed by Western blotting using the indicated antibodies. Click here for file

Additional file 3: Figure S3 Over-expression of AnxA6 in HCC1806 enhances the expression of EGFR but inhibits receptor activation and anchorage-independent growth. (A) Control (HCC1806-EV) and AnxA6 over-expressing HCC1806 (HCC1806-AnxA6) cells were grown to 70% confluency and serum-starved for 24 h. Cells were then treated with EGF for 0–90 min, and whole cell lysates were analyzed by western blotting using the indicated antibodies. End.AnxA6 = endogenous AnxA6 (B) Densitometric analysis of AnxA6 and EGFR protein expression. Expression levels in control and AnxA6 over-expressing HCC1806 cells were normalized to GAPDH. Bars represent AnxA6 or EGFR protein expression ± s.d. from three independent experiments relative to the levels in control cells. (C) Densitometric analysis of activated EGFR. Points represent phospho-EGFR remaining at the indicated times from a representative experiment. (D) Densitometric analysis of activated ERK1/2. Points represent phospho-ERK1/2 levels at the indicated times from a representative experiment. (E) 3D Matrigel growth assays. Control and AnxA6 over-expressing HCC1806 cells (5 × 10^3^ cells/assay) were cultured in 3D matrigel cultures for up to 10 days. Digital images of the colonies were captured with a digital camera (x10 magnification). Click here for file

## References

[B1] Buzhynskyy N Golczak M Lai-Kee-Him J Lambert O Tessier B Annexin-A6 presents two modes of association with phospholipid membranes. A combined QCM-D, AFM and cryo-TEM study J Struct Biol 2009 12 107 116 10.1016/j.jsb.2009.03.007 19306927

[B2] Song G Campos B Wagoner LE Dedman JR Walsh RA Altered cardiac annexin mRNA and protein levels in the left ventricle of patients with end-stage heart failure J Mol Cell Cardiol 1998 12 443 451 10.1006/jmcc.1997.0608 9515022

[B3] Ueng KC Lin CS Yeh HI Wu YL Liu RH Downregulated cardiac annexin VI mRNA and protein levels in chronically fibrillating human atria Cardiology 2008 12 208 216 10.1159/000106685 17726323

[B4] Francia G Mitchell SD Moss SE Hanby AM Marshall JF Identification by differential display of annexin-VI, a gene differentially expressed during melanoma progression Cancer Res 1996 12 3855 3858 8752144

[B5] Sakwe AM Koumangoye R Guillory B Ochieng J Annexin A6 contributes to the invasiveness of breast carcinoma cells by influencing the organization and localization of functional focal adhesions Exp Cell Res 2011 12 823 837 10.1016/j.yexcr.2010.12.008 21185831 PMC3049817

[B6] Song G Harding SE Duchen MR Tunwell R O'Gara P Altered mechanical properties and intracellular calcium signaling in cardiomyocytes from annexin 6 null-mutant mice FASEB J 2002 12 622 624 11919174 10.1096/fj.01-0892fje

[B7] Monastyrskaya K Babiychuk EB Hostettler A Wood P Grewal T Plasma membrane-associated annexin A6 reduces Ca2+ entry by stabilizing the cortical actin cytoskeleton J Biol Chem 2009 12 17227 17242 10.1074/jbc.M109.004457 19386597 PMC2719360

[B8] Wu CY Taneyhill LA Annexin a6 modulates chick cranial neural crest cell emigration PLoS One 2012 12 e44903 10.1371/journal.pone.0044903 22984583 PMC3439457

[B9] Minashima T Small W Moss SE Kirsch T Intracellular modulation of signaling pathways by annexin A6 regulates terminal differentiation of chondrocytes J Biol Chem 2012 12 14803 14815 10.1074/jbc.M111.297861 22399299 PMC3340232

[B10] Cheang MC Voduc D Bajdik C Leung S McKinney S Basal-like breast cancer defined by five biomarkers has superior prognostic value than triple-negative phenotype Clin Cancer Res 2008 12 1368 1376 10.1158/1078-0432.CCR-07-1658 18316557

[B11] Rakha EA Elsheikh SE Aleskandarany MA Habashi HO Green AR Triple-negative breast cancer: distinguishing between basal and nonbasal subtypes Clin Cancer Res 2009 12 2302 2310 10.1158/1078-0432.CCR-08-2132 19318481

[B12] Tan DS Marchio C Jones RL Savage K Smith IE Triple negative breast cancer: molecular profiling and prognostic impact in adjuvant anthracycline-treated patients Breast Cancer Res Treat 2008 12 27 44 10.1007/s10549-007-9756-8 17922188

[B13] Arteaga C Targeting HER1/EGFR: a molecular approach to cancer therapy Semin Oncol 2003 12 3 14 12840796

[B14] Baselga J The EGFR as a target for anticancer therapy–focus on cetuximab Eur J Cancer 2001 12 Suppl 4 S16 S22 11597400 10.1016/s0959-8049(01)00233-7

[B15] Nicholson RI Gee JM Harper ME EGFR and cancer prognosis Eur J Cancer 2001 12 Suppl 4 S9 S15 11597399 10.1016/s0959-8049(01)00231-3

[B16] Mukhopadhyay P Lakshmanan I Ponnusamy MP Chakraborty S Jain M MUC4 overexpression augments cell migration and metastasis through EGFR family proteins in triple negative breast cancer cells PLoS One 2013 12 e54455 10.1371/journal.pone.0054455 23408941 PMC3569463

[B17] Babiychuk EB Draeger A Annexins in cell membrane dynamics. Ca(2+)-regulated association of lipid microdomains J Cell Biol 2000 12 1113 1124 10.1083/jcb.150.5.1113 10973999 PMC2175252

[B18] Cornely R Rentero C Enrich C Grewal T Gaus K Annexin A6 is an organizer of membrane microdomains to regulate receptor localization and signalling IUBMB Life 2011 12 1009 1017 10.1002/iub.540 21990038

[B19] Grewal T Evans R Rentero C Tebar F Cubells L Annexin A6 stimulates the membrane recruitment of p120GAP to modulate Ras and Raf-1 activity Oncogene 2005 12 5809 5820 10.1038/sj.onc.1208743 15940262

[B20] Vila de Muga S Timpson P Cubells L Evans R Hayes TE Annexin A6 inhibits Ras signalling in breast cancer cells Oncogene 2009 12 363 377 10.1038/onc.2008.386 18850003

[B21] Schmitz-Peiffer C Browne CL Walker JH Biden TJ Activated protein kinase C alpha associates with annexin VI from skeletal muscle Biochem J 1998 12 Pt 2 675 681 9480874 10.1042/bj3300675 PMC1219189

[B22] Chow A Davis AJ Gawler DJ Identification of a novel protein complex containing annexin VI, Fyn, Pyk2, and the p120(GAP) C2 domain FEBS Lett 2000 12 88 92 10.1016/S0014-5793(00)01252-7 10708762

[B23] Koese M Rentero C Kota BP Hoque M Cairns R Annexin A6 is a scaffold for PKCalpha to promote EGFR inactivation Oncogene 2013 12 2858 2872 10.1038/onc.2012.303 22797061

[B24] Babiychuk EB Palstra RJ Schaller J Kampfer U Draeger A Annexin VI participates in the formation of a reversible, membrane-cytoskeleton complex in smooth muscle cells J Biol Chem 1999 12 35191 35195 10.1074/jbc.274.49.35191 10575003

[B25] Locate S Colyer J Gawler DJ Walker JH Annexin A6 at the cardiac myocyte sarcolemma–evidence for self-association and binding to actin Cell Biol Int 2008 12 1388 1396 10.1016/j.cellbi.2008.08.009 18782625

[B26] Ortega D Pol A Biermer M Jackle S Enrich C Annexin VI defines an apical endocytic compartment in rat liver hepatocytes J Cell Sci 1998 12 Pt 2 261 269 9405315 10.1242/jcs.111.2.261

[B27] Pons M Grewal T Rius E Schnitgerhans T Jackle S Evidence for the Involvement of annexin 6 in the trafficking between the endocytic compartment and lysosomes Exp Cell Res 2001 12 13 22 10.1006/excr.2001.5268 11525635

[B28] Domon MM Matar G Strzelecka-Kiliszek A Bandorowicz-Pikula J Pikula S Interaction of annexin A6 with cholesterol rich membranes is pH-dependent and mediated by the sterol OH J Colloid Interface Sci 2010 12 436 441 10.1016/j.jcis.2010.03.015 20363475

[B29] Sztolsztener ME Strzelecka-Kiliszek A Pikula S Tylki-Szymanska A Bandorowicz-Pikula J Cholesterol as a factor regulating intracellular localization of annexin A6 in Niemann-Pick type C human skin fibroblasts Arch Biochem Biophys 2009 12 221 233 19900398 10.1016/j.abb.2009.11.001

[B30] Sorkin A Duex JE Quantitative analysis of endocytosis and turnover of epidermal growth factor (EGF) and EGF receptor Curr Protoc Cell Biol 2010 12 15 14 20235100 10.1002/0471143030.cb1514s46 PMC2878126

[B31] Sorkin A Goh LK Endocytosis and intracellular trafficking of ErbBs Exp Cell Res 2008 12 3093 3106 18793634 10.1016/j.yexcr.2008.07.029 PMC2605728

[B32] Xie J Qian L Wang Y Rose CM Yang T Novel biphasic traffic of endocytosed EGF to recycling and degradative compartments in lacrimal gland acinar cells J Cell Physiol 2004 12 108 125 10.1002/jcp.10458 14978740

[B33] Irwin ME Mueller KL Bohin N Ge Y Boerner JL Lipid raft localization of EGFR alters the response of cancer cells to the EGFR tyrosine kinase inhibitor gefitinib J Cell Physiol 2011 12 2316 2328 10.1002/jcp.22570 21660955 PMC3103760

[B34] Liu Y Sun R Wan W Wang J Oppenheim JJ The involvement of lipid rafts in epidermal growth factor-induced chemotaxis of breast cancer cells Mol Membr Biol 2007 12 91 101 10.1080/10929080600990500 17453416

[B35] Lu YC Chen HC Involvement of lipid rafts in adhesion-induced activation of Met and EGFR J Biomed Sci 2011 12 78 10.1186/1423-0127-18-78 22032640 PMC3244112

[B36] Gyorffy B Benke Z Lanczky A Balazs B Szallasi Z Recurrence Online: an online analysis tool to determine breast cancer recurrence and hormone receptor status using microarray data Breast Cancer Res Treat 2010 12 1025 1034 10.1007/s10549-011-1676-y 21773767

[B37] Blanco MA Aleckovic M Hua Y Li T Wei Y Identification of staphylococcal nuclease domain-containing 1 (SND1) as a Metadherin-interacting protein with metastasis-promoting functions J Biol Chem 2011 12 19982 19992 10.1074/jbc.M111.240077 21478147 PMC3103372

[B38] Chakrabarti R Hwang J Andres Blanco M Wei Y Lukacisin M Elf5 inhibits the epithelial-mesenchymal transition in mammary gland development and breast cancer metastasis by transcriptionally repressing Snail2 Nat Cell Biol 2012 12 1212 1222 10.1038/ncb2607 23086238 PMC3500637

[B39] Noordermeer SM Wennemers M Bergevoet SM van der Heijden A Tonnissen E Expression of the BRCA1 complex member BRE predicts disease free survival in breast cancer Breast Cancer Res Treat 2012 12 125 133 10.1007/s10549-012-2122-5 22706632 PMC3413819

[B40] Chi S Cao H Wang Y McNiven MA Recycling of the epidermal growth factor receptor is mediated by a novel form of the clathrin adaptor protein Eps15 J Biol Chem 2011 12 35196 35208 10.1074/jbc.M111.247577 21832070 PMC3186422

[B41] Lill NL Sever NI Where EGF receptors transmit their signals Sci Signal 2012 12 pe41 23012653 10.1126/scisignal.2003341 PMC3507515

[B42] Sousa LP Lax I Shen H Ferguson SM De Camilli P Suppression of EGFR endocytosis by dynamin depletion reveals that EGFR signaling occurs primarily at the plasma membrane Proc Natl Acad Sci USA 2012 12 4419 4424 10.1073/pnas.1200164109 22371560 PMC3311323

[B43] Hofman EG Ruonala MO Bader AN van den Heuvel D Voortman J EGF induces coalescence of different lipid rafts J Cell Sci 2008 12 2519 2528 10.1242/jcs.028753 18628305

[B44] Cubells L Vila de Muga S Tebar F Wood P Evans R Annexin A6-induced alterations in cholesterol transport and caveolin export from the Golgi complex Traffic 2007 12 1568 1589 10.1111/j.1600-0854.2007.00640.x 17822395 PMC3003291

[B45] Domon MM Besson F Bandorowicz-Pikula J Pikula S Annexin A6 is recruited into lipid rafts of Niemann-Pick type C disease fibroblasts in a Ca2 + −dependent manner Biochem Biophys Res Commun 2012 12 192 196 10.1016/j.bbrc.2010.12.138 21216236

[B46] Anders CK Carey LA Biology, metastatic patterns, and treatment of patients with triple-negative breast cancer Clin Breast Cancer 2009 12 Suppl 2 S73 S81 19596646 10.3816/CBC.2009.s.008 PMC2919761

