# Supplementary figures and images for: Reduced annexin A6 expression promotes the degradation of activated epidermal growth factor receptor and sensitizes invasive breast cancer cells to EGFR-targeted tyrosine kinase inhibitors
Source: Mol Cancer. 2013 Dec 19;12:167. doi: 10.1186/1476-4598-12-167 (PMC3922904; doi:10.1186/1476-4598-12-167)

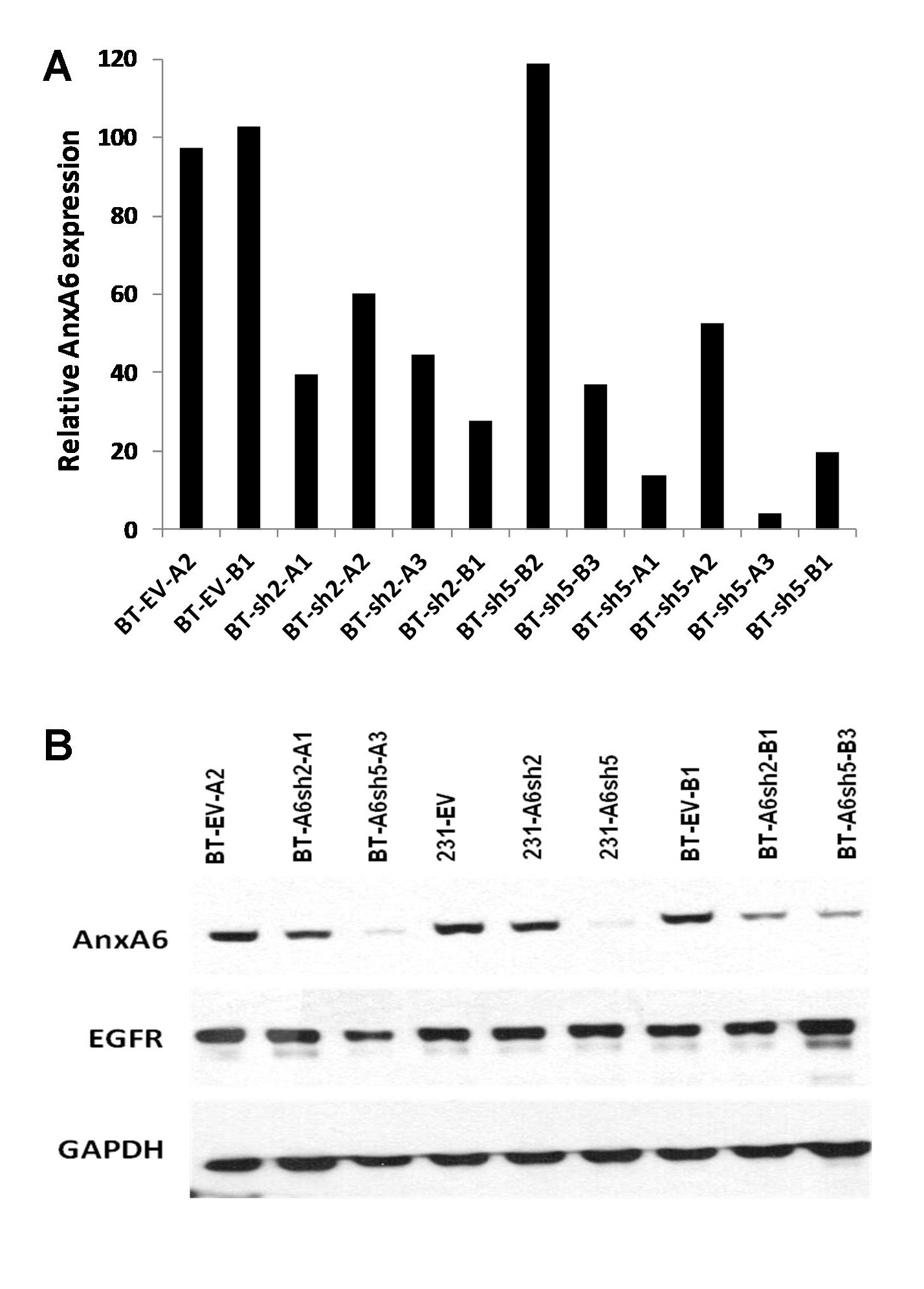

Supplement: Additional file 1: Figure S1 — Down regulation of AnxA6 in BT-549 and MDA-MB-231 breast cancer cells. (A) BT-549 and MDA-MB-231 cell lines were transfected with shRNAs in pGIPZ and cloned as described in “Materials and methods”. Whole cell lysates from the isolated clones generated from two distinct shRNAs designated A6sh2 and A6sh5 were analyzed by Western blotting. Densitometric analysis of the expression of AnxA6 expression relative to GAPDH is presented. B) Whole cell lysates from the selected clones were analyzed by western blotting using antibodies against AnxA6, EGFR and GAPDH. [file 1476-4598-12-167-S1.jpeg]

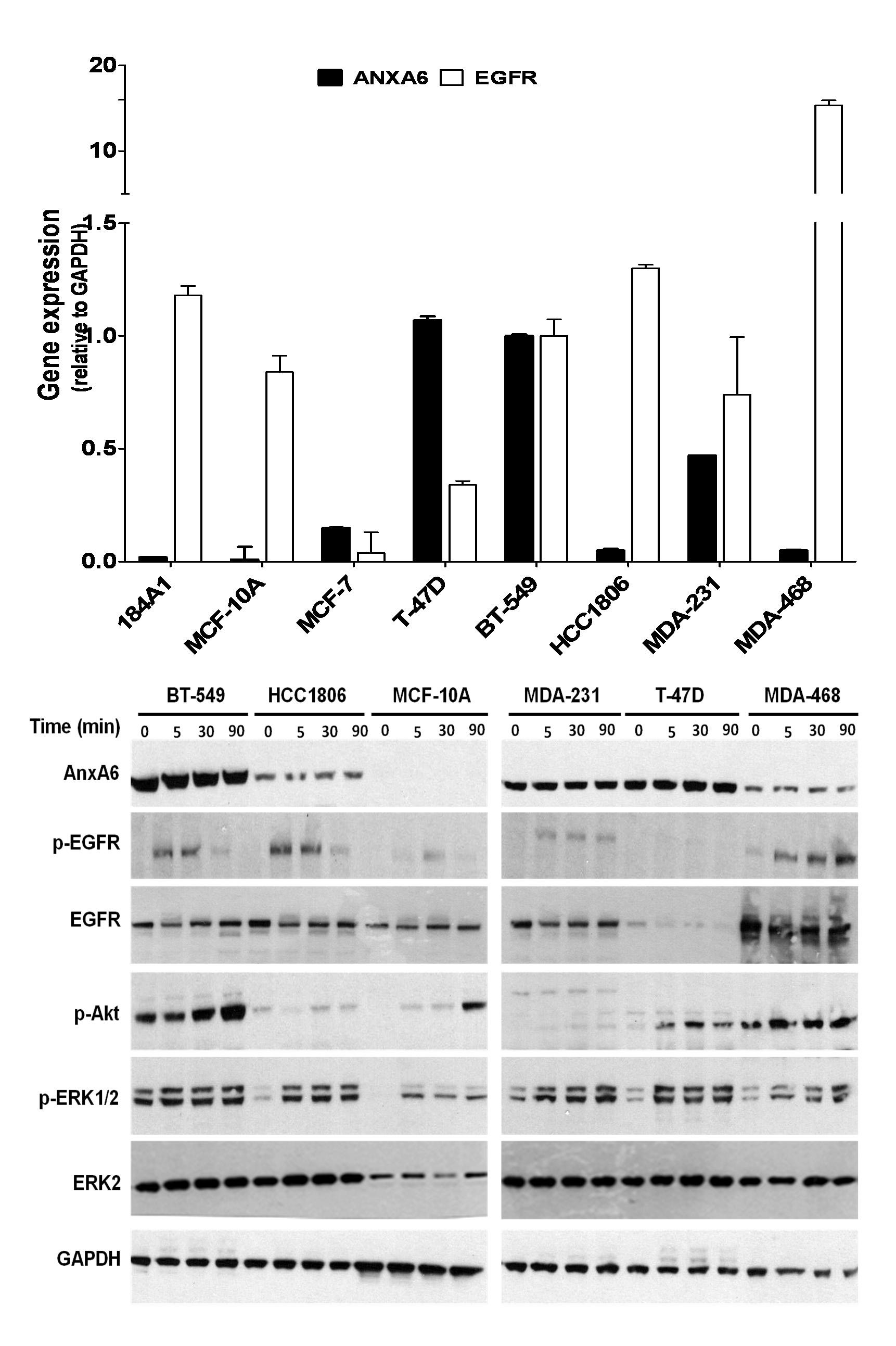

Supplement: Additional file 2: Figure S2 — Differential expression of AnxA6 and EGF-induced activation of EGFR in normal breast epithelial and breast cancer cells A) mRNA levels of AnxA6 and EGFR in normal breast epithelial and breast cancer cells. Equal amounts (1 μg) of total RNA extracted from the indicated cell lines were used for the first strand synthesis and quantitative PCR was programmed with 10% of the first strand reactions. Bars represent gene expression levels normalized to GAPDH ± s.d. from three independent determinations. (B) AnxA6 expression and EGF-induced activation of EGFR and downstream signaling in normal and breast carcinoma cell lines. The indicated cell lines were grown to 70% confluency, followed by serum starvation for 24 h. Cells were then treated with EGF for 0–90 min and harvested by scrapping in ice-cold PBS. Equal amounts of whole cell lysates were separated in 4-12% polyacrylamide gels under reducing conditions and analyzed by Western blotting using the indicated antibodies. [file 1476-4598-12-167-S2.jpeg]

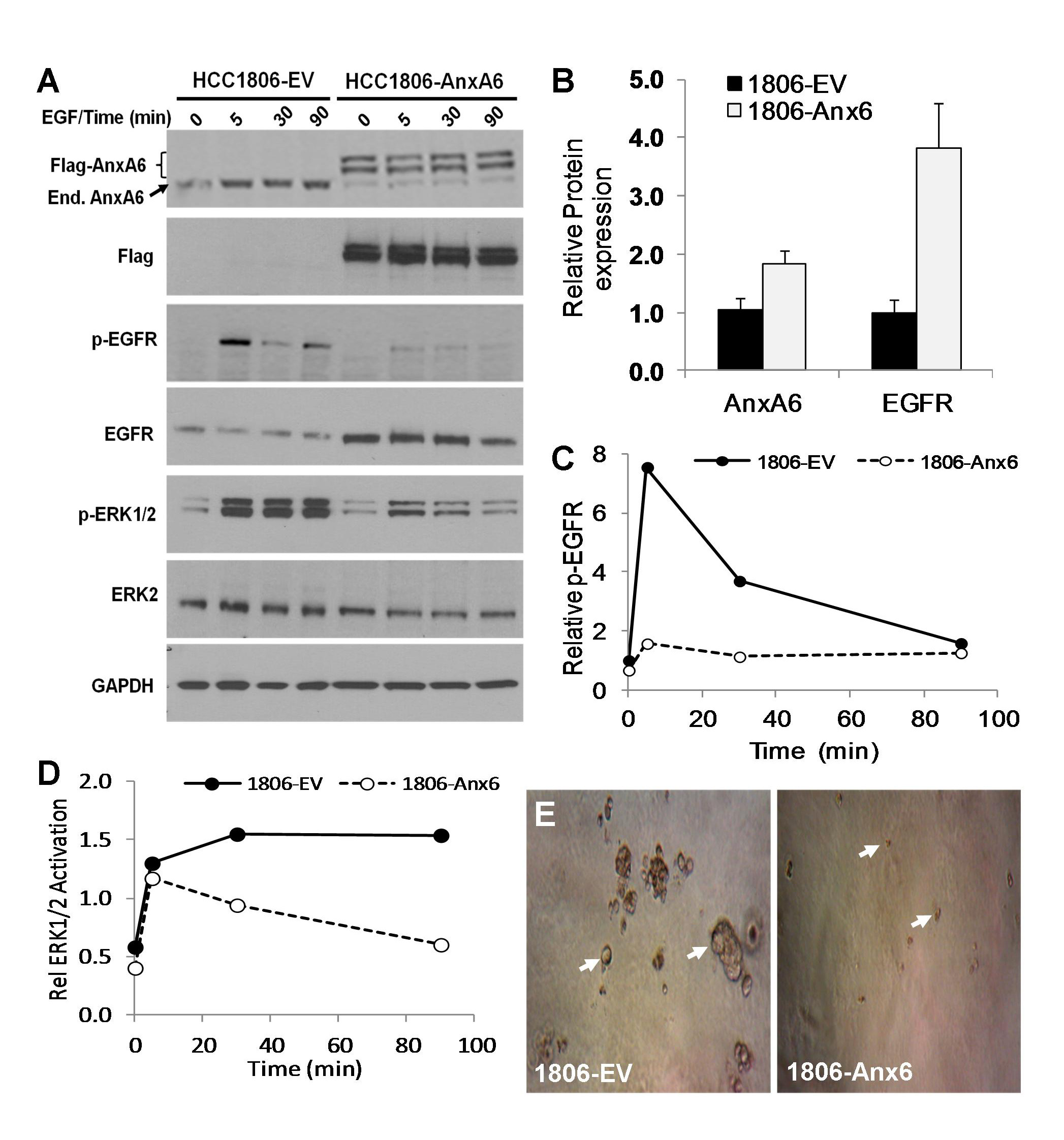

Supplement: Additional file 3: Figure S3 — Over-expression of AnxA6 in HCC1806 enhances the expression of EGFR but inhibits receptor activation and anchorage-independent growth. (A) Control (HCC1806-EV) and AnxA6 over-expressing HCC1806 (HCC1806-AnxA6) cells were grown to 70% confluency and serum-starved for 24 h. Cells were then treated with EGF for 0–90 min, and whole cell lysates were analyzed by western blotting using the indicated antibodies. End.AnxA6 = endogenous AnxA6 (B) Densitometric analysis of AnxA6 and EGFR protein expression. Expression levels in control and AnxA6 over-expressing HCC1806 cells were normalized to GAPDH. Bars represent AnxA6 or EGFR protein expression ± s.d. from three independent experiments relative to the levels in control cells. (C) Densitometric analysis of activated EGFR. Points represent phospho-EGFR remaining at the indicated times from a representative experiment. (D) Densitometric analysis of activated ERK1/2. Points represent phospho-ERK1/2 levels at the indicated times from a representative experiment. (E) 3D Matrigel growth assays. Control and AnxA6 over-expressing HCC1806 cells (5 × 10^3^ cells/assay) were cultured in 3D matrigel cultures for up to 10 days. Digital images of the colonies were captured with a digital camera (x10 magnification). [file 1476-4598-12-167-S3.jpeg]
